# 

**Published:** 1816-07

**Authors:** 


### THE
### Medico-Chirurgical
### JOURNAL AND REVIEW.

CONTAINING

I. Select Original Communications.

II. Comprehensive Analytical Review of all important Medical Publications; presenting a fair Epitome of the Medical Literature of the Day.

III. Select Translations from Foreign Medical Journals.

IV. Medical Miscellanies, Foreign and Domestic; Anonymous Communications, &c. &c.

S.G.O. INDEXED

CONDUCTED BY

WILLIAM SHEARMAN, M. D.

Licentiate of the Royal College of Physicians, and Physician to the London Dispensary;

JAMES JOHNSON, M. D.

Surgeon to His Royal Highness the Duke of Clarence's Household; and Author of "The Influence of Tropical Climates on European Constitutions."

AND

SHIRLEY PALMER, M. D.

Of the University of Glasgow, and Member of the Royal College of Surgeons, London.

OMNIA VINCIT LABOR SI LABOR IPSE VOLUPTAS.

SURGEON GEN'LS OFFICE LIBRARY. 63385

VOL. II.

FROM JUNE TO DECEMBER, 1816.

July –

*LONDON.*

PRINTED FOR THE PROPRIETORS,

BY WILLIAM THORNE, RED LION COURT, FLEET STREET,

To whom Communications, addressed to the Editors, are requested to be sent.

Sold by BALDWIN, CRADDOCK, and JOY, Pater-Noster Row; HIGHLEY and SON, and T. & G. UNDERWOOD, Fleet Street; J. CALLOW, Crown Court, Soho; COX and SON, Borough; J. ANDERSON, West Smithfield; W. BLACKWOOD, Edinburgh; J. CUMMING, Dublin; and may be had also by applying to the Clerks of the Roads, at the General Post Office.

W
1
ME 75H

### ADVERTISEMENT.

THE Second Volume of the MEDICO-CHIRURGICAL JOURNAL has now reached its close; and, if a retrospect of the year's labour be satisfactory to the Editors, the prospect before them is not less fair and encouraging.—In circulation, as in character, their work has been steadily progressive from its commencement. The most honourable marks of public approbation and respect have been received by the Editors from all parts of the island. Testimonies like these, might have warmed with gratitude, or inflamed with vanity, the hearts of men less conscious than themselves of having laboured strenuously to deserve them. Upon the minds of the Editors they exercise an influence, deeper in utility as more elevated and pure in principle: they operate as powerful incentives to unremitting perseverance and increased exertion.

To explain the views and objects of the MEDICO-CHIRURGICAL JOURNAL were now as useless as to avow the principles on which it is, in future, to be conducted. Upon this point, the two volumes already before the public are fraught with sufficient evidence. In unwearied pursuit of objects thus dignified—in fearless and unconquerable adherence to principles thus independent, the Editors have solemnly pledged themselves to stand or fall. Failure with these, they would consider honourable; the success, purchased by their sacrifice, worthless and disgraceful.

Of the adequacy of their resources in prosecuting their work, the Editors can now speak with implicit confidence. Means, which a year's expenditure could neither exhaust nor sensibly impair, must spring from no precarious or despicable source. In fact, like the well-employed talent of Eastern parable, they have only increased by use and culture. In that department which alone relies for existence on public contribution, the Editors have endeavoured, by dint of extraordinary exertion, during the last year, to provide—and render themselves independent for the next; nor have they tried in vain. Such a mass of ORIGINAL MATTER has been accumulated by them, as for magnitude, and perhaps, also for general importance, no Journal of Medicine, it may be confidently asserted, has ever yet possessed at one time.

The Editors, nevertheless, solicit from the Public valuable contributions on all subjects connected with medical science. They solicit, however, not in the importunate tone of perishing necessity, but in the manly language of men conscious of their independence, and confident in their own resources; and who, steadily rejecting trifles, have determined to accept such donations only as are calculated to do honour alike to the hand which presents, and to that which receives them.

SHEARMAN,
JOHNSON,
PALMER.

London, December 1, 1816.

☞ Numerous complaints of neglect or disappointment in the supply of the MEDICO-CHIRURGICAL JOURNAL have, during the few last months, reached the Editors. Gentlemen are, therefore, requested to be very precise in their orders for the work; and, in the event of encountering any difficulty, to make direct application to the Editors, Dr. Shearman, Owen's Place, Goswell-Street Road; Dr. Johnson, St. George's Square, Portsea; Dr. Palmer, Tamworth, Staffordshire; or to Mr. Thorne, Printer, Red Lion Court, Fleet Street; when every exertion will be made to trace the irregularity to its source, and to obviate its recurrence.

THE

### Medico-Chirurgical

### Journal and Review.

VOL. II.] JULY, 1816. [NO. 7.

### ADVERTISEMENT.

*IN entering upon the Second Volume of "The Medico-Chirurgical Journal and Review," the Editors beg leave to offer a few remarks on the objects and execution of their work.*

*The* Original Department *of their First Volume, although containing fewer valuable papers than they had hoped to see it comprehend, may justly claim, it is presumed, the character of respectability. To preserve it from the pollution of trash, and the contemptible spirit of acrid and unmeaning controversy, will form an object of their strenuous exertions. Circumstances have recently arisen, which must, for a time, exercise an unfavourable influence upon this department. But the Editors look forward with confidence to that increase both in the number and value of contributions, which the independence of their principles, and unconquerable zeal for the good cause, cannot fail to attract from a British Public.*

*Of the* Review Department, *the Volume, recently concluded, displays a specimen, which the Editors will fearlessly assume as the standard of their future performance. This constitutes the strength, the pillar, the main object of their labours; and simply by its merits are they content that the fate*

*and character of the work shall be decided. A system of review, so comprehensive, both with respect to the number of books which it includes and the style of analysis, has never yet been attempted by any Journal of Medicine, British or Continental. The advantages of which such a system, steadily pursued, must be productive to the numerous class of busy or less opulent practitioners, are too obvious to require comment. The Editors calculate upon being able to present, annually, an analysis, equally diffuse, of between thirty and forty volumes.*

*They have, at the same time, to regret that the* Selection Department *has not yet acquired the value and extent which they aspire to give it. This defect is principally attributable to some obstructions which they have unexpectedly experienced in the supply of Foreign Journals. Arrangements, however, have recently been concluded, by which all the more important periodical works on Medicine, afforded by Germany, France, and Italy, will be regularly received: and henceforth from twelve to fifteen Foreign Journals will be made tributary to this department. In the constitution of it, Forensic Medicine, Medical Botany, and Chemistry, will occupy a conspicuous rank. But the Editors beg to observe, that the botanical, chemical, and other accessory sciences, will be cultivated only in their more obvious and intimate relations with practical medicine.*

*Lastly, they will endeavour to give to the* Miscellanies *additional variety and value; and they trust that the door, being thus thrown open to anonymous communications, many an interesting fugitive will be ushered in, which otherwise had perished in the tumult of practice, or, ere long, been swept away by the stream of oblivion.*

SHEARMAN.
London, July 1st, 1816. JOHNSON.
PALMER.

CORRESPONDENCE.

Communications are received from Mr. Bailey, Mr. Dewar, Mr. Edw. Doughty, Mr. Howship, and Dr. Von Embden.

